# Clinical Cases

**Published:** 1860-10

**Authors:** N. S. Davis


### CLINICAL CASES.

BY N. S. DAVIS, M. D., &c.

### *Alcohol in the Dilirium of Typhoid Fever.*

Mr. S——; a middle aged man, was attacked with the ordinary symptoms of Typhoid Fever, and after running a course of moderate severity, under the care of an intelligent and judicious practitioner of this city, canvalescence ensued at the end of the third week. Under the use of mild tonics and suitable nourishment he continued apparently convalescent for several days; when the symptoms of fever returned. His pulse became quick; skin dry; mind wandering; slight sub-sultus; and bowels loose. The intestinal discharges were thin and of a grey or ash color, and the urine scanty.

His physician gave him a few doses of Dover's Powder with a small quantity of calomel, which had the effect to change the color of the evacuations, but not their frequency. He then gave small doses of nitrate of silver, and a Dover's Powder at night to procure rest, and endeavored to support the patient by nourishment and wine or porter. The patient, however, continued to get worse. He became more wakeful, more delirious, with more sub-sultus, a feebler pulse, and a continuance of the same looseness of the bowels. Being called in consultation, at this stage of the disease, I did not hesitate to sanction the continuance of nourishment with an increased quantity of stimulants, under the hope that the latter would controll the delirium and sub-sultus as claimed by Dr. Todd and many others. To counteract an evident tendency to ulceration in the aggregated glands of the ilium, an emulsion of oil of turpentine and tincture of opium was ordered, and regularly administrated. This treatment was continued three days, during which time the intestinal discharges became less frequent, the skin less dry; but the pulse continued frequent and feeble, and the force of the heart's action was decidedly impaired. The vigilance and delirium also increased. It was evident from the weakness of the impulse of the heart, the absence of any noted alterations in the pupil of the eye, &c., that the delirium in this case was functional, and that all the symptoms were such, as Dr. Todd and others, claim to be under the control of alcoholic stimulants. Hence these stimulants, in the form of porter and brandy had been gradually increased, until during the last twenty-four hours, he had taken of the latter alone, more than a pint; but without the slightest amelioration of the symptoms. On applying the ear to the chest, I found over the middle lobe of the right lung a moderate sub-mucous rhonchus, with diminished respiratory murmur over the lower lobes on both sides.

Being satisfied that the alcoholic stimulants were doing no good, and that passive engorgement of the lungs had already commenced in addition to the previous grave symptoms, I advised his attending physician to omit all the alcoholic stimulants, and to give the following:

| | | |
|---|---|---|
| ℞. | Strychnine, | 1 gr. |
| | Nitric Acid, | ʒ i. |
| | Tinct. Opium, | ʒ ii. |
| | Water, | ℥ ii. |

Mix, and give a teaspoonful every 4 hours in sweetened water; also one of the following powders two hours after each dose of the strychnine solution, viz:

| | | |
|---|---|---|
| ℞. | Pulv. Doveri, | ʒ i. |
| | Pulv. G. Camph., | 12 grs. |

Mix, and divide into six powders. He was also well supported with beef-tea or soup, salted with chlorate of potassa. During the first twelve hours after the stimulants were discontinued, he was very restless and delirious; he then became quiet and slept almost continuously for five or six hours, being aroused only to take a few spoonfuls of nourishment at suitable intervals. On awaking, he appeared feeble, but less delirious than for several days previous. The evacuations from the bowels, though not frequent, continued thin and greyish brown. On this account 10 drops of oil of turpentine were given in the form of an emulsion in place of the powders, one of the latter to be given in the evening only. At the end of forty-eight hours after commencing the use of the strychnine, the patient was entirely free from delirium, and all his symptoms improved. Soon after that, however, he complained of severe stiffness in the muscles of the neck, with occasional sudden muscular twitchings, and the discontinuance of the remedy was deemed advisable. In its place he took, every four hours, a pill containing nitras argent. one-third of a grain and opium half a grain, and continued the turpentine emulsion, together with the same nourishment as before. Under a continuance of these remedies he fully convalesced in about four days.

---

*Typhoid Fever with Paralysis of the Muscles of Deglutition.*

Miss C———, a Norwegian girl, aged 13 years, had been sick and under the care of a physician for little more than two weeks, before she came under my care. From what I could learn, she had been laboring under the ordinary symptoms of a grave case of Typhoid Fever; and had been treated with diffusable stimulants moderately, and a fair supply of nourishment. I found her with a small feeble pulse, 110 per minute; skin dry but not hot; face rather suffused with a dark or venous redness; extremeties cool; bowels quiet; and total inability either to swallow, speak, or protrude her tongue. She was conscious and apparently rational, and made an effort to answer a question, but could not command the necessary muscular action. The mother said she had not swollowed a drop or spoken a word during the past two days. As it was evident that the force of the heart's action, and all the other important functions were failing under the combined influence of disease and starvation, I ordered the following:

| | | |
|---|---|---|
| ℞ | Strychnine, | 1 gr. |
| | Nitric Acid, | 20 gtts. |
| | Tict. Opii, | ʒ ii. |
| | Water, | ℥ ii. |

Mix one teaspoonful, to be put into a teacupful of beef-tea, and injected into the rectum, and repeated every six hours. These injections were wholly retained and absorbed. This course was continued faithfully for two days; during which there was a decided improvement of the pulse, the color of the skin, and the expression of countenance. Near the end of the second day, she succeeded in swallowing two teaspoonfuls of milk.

On the following morning, she took several spoonfuls of milk with much greater facility. Still she could not speak. I now ordered the same medicine to be given by the mouth, in doses of 30 drops, every four hours; and a gill of sweet milk half way between the doses of the medicine.

On the fourth day after she came under my care, she could swallow quite easily, and speak, but not distinctly. Several evacuations from the bowels had also occurred, the discharges being liquid, and of a brown color. On this account I extended the interval between the doses of Strychnine, to six hours, and gave of oil of Turpentine and Tinct. of Opium, each eight drops, half way between. A liberal supply of milk was allowed for nourished, with the addition of rice. Under this treatment she continued to improve, and was fully convalescent at the end of ten days; which was about twenty-six days from the time her fever commenced.

*Remark.*—The strychnine was given in both of the preceding cases, from its known efficacy in increasing innervation and muscular contractility, and thereby increasing the force of the heart's action, and sustaining the functions of circulation and respiration.

